# Costs Associated With Modifiable Risk Factors in Ventral and Incisional Hernia Repair

**DOI:** 10.1001/jamanetworkopen.2019.16330

**Published:** 2019-11-27

**Authors:** Ryan Howard, Michael Thompson, Zhaohui Fan, Michael Englesbe, Justin B. Dimick, Dana A. Telem

**Affiliations:** 1 Department of Surgery, University of Michigan Health System, Ann Arbor; 2 Department of Cardiac Surgery, University of Michigan Medical School, Ann Arbor; 3 Michigan Value Collaborative, Ann Arbor; 4 Center for Healthcare Outcomes and Policy, Ann Arbor, Michigan

## Abstract

**Importance:**

Ventral and incisional hernia repair (VIHR) is an extremely common operation, after which complications are also fairly common. A number of preoperative risk factors are known to contribute to increased complications after surgical repair; however, the individual relative association of these risk factors with adverse outcomes and increased spending is unclear. Quantifying the association of individual risk factors may help surgeons implement targeted surgical optimization, improve outcomes, and reduce spending.

**Objective:**

To identify the attributable association of modifiable risk factors for adverse outcomes after VIHR on outcomes and episode-of-care payments.

**Design, Setting, and Participants:**

This cross-sectional study was performed using a population-based sample of adult patients and episode spending data from January 1, 2012, to December 31, 2018, from a statewide multipayer registry. A multilevel mixed-effects logistic regression model was used to examine the contribution of patient-specific risk factors to adverse outcomes. Attributable risk and population attributable risk fraction were calculated to estimate the additional spending attributable to individual risk factors. Data were analyzed from April 2018 to September 2018.

**Main Outcomes and Measures:**

Any complications, serious complication, discharge not to home, 30-day emergency department utilization, and 30-day readmission. Episode-of-care spending was calculated for these outcomes.

**Results:**

This study included 22 664 patients (median [interquartile range] age, 55 [44-64] years; 10 496 [46.3%] women) undergoing VIHR with identified significant preoperative risk factors. Fourth-quartile body mass index (BMI), calculated as weight in kilograms divided by height in meters squared and defined as a mean (SD) BMI of 43 (6), was associated with increased risk of any complication (odds ratio [OR], 1.64; 95% CI, 1.30-2.06; *P* < .001) and serious complication (OR, 1.67; 95% CI, 1.22-2.31; *P* = .002). Insulin-dependent diabetes was associated with increased risk of any complication (OR, 1.34; 95% CI, 1.03-1.73; *P* = .03), serious complication (OR, 1.51; 95% CI, 1.08-2.12; *P* = .02), discharge not to home (OR, 1.49; 95% CI, 1.12-1.98; *P* = .005), and 30-day readmission (OR, 1.68; 95% CI, 1.32-2.14; *P* < .001). Median (interquartile range) additional episode spending for any complication was $9934 ($9224-$11 851), of which $1304 ($1208-$1552) was attributable to fourth-quartile BMI. Median (interquartile range) additional episode spending for a serious complication was $26 648 ($20 632-$33 166), of which $3638 ($2827-$4544) was attributable to fourth-quartile BMI, $650 ($495-$796) was attributable to insulin-dependent diabetes, and $567 ($433-$696) was attributable to unhealthy alcohol use.

**Conclusions and Relevance:**

In this cross-sectional study, modifiable risk factors, such as obesity, insulin-dependent diabetes, and unhealthy alcohol use, were associated with adverse outcomes after VIHR. These factors were significantly associated with increased health care spending; therefore, preoperative optimization may improve outcomes and decrease episode-of-care costs.

## Key Points

**Question:**

What is the attributable association of modifiable preoperative risk factors with clinical outcomes and health care spending after ventral and incisional hernia repair (VIHR)?

**Findings:**

In this cross-sectional analysis of 22 664 adult patients undergoing VIHR, morbid obesity, insulin-dependent diabetes, and unhealthy alcohol use were significantly associated with adverse outcomes. Additional spending for a serious complication after surgery was $26 648, of which $3638 was associated with morbid obesity, $650 was associated with insulin-dependent diabetes, and $567 was associated with unhealthy alcohol use.

**Meaning:**

Quantifying the association of individual risk factors with adverse outcomes after VIHR may help surgeons develop targeted interventions to reduce complications and surgical spending.

## Teaser

This cross-sectional study examines the attributable association of modifiable risk factors for adverse outcomes after hernia repair and increased health care spending associated with these adverse outcomes.

## Introduction

More than 350 000 ventral and incisional hernia repairs (VIHRs) are performed each year in the United States.^1^ The annual health care spending associated with these operations exceeds $3 billion. Unfortunately, a significant proportion of VIHR are associated with complications, with 30-day readmission rates of 5%, surgical site infection rates of 13%, and recurrence rates as high as 63%.^2,3^ Although variation in operative approach and technique has been shown to affect outcomes, it is also well known that a number of patient comorbidities can significantly affect postoperative mortality and morbidity.^4^ Diabetes, obesity, and low functional status have been shown to increase short-term wound infection and readmission rate, as well as long-term hernia recurrence and need for reoperation.^5,6,7^ The increased costs associated with these modifiable patient risk factors have been reported to exceed $80 000 per patient.^8^

Forgoing operative VIHR in high-risk patients avoids postoperative complications, but it is associated with decreased functional status and poor quality of life and exposes patients to the risk of emergency VIHR.^9,10^ Consequently, there is increasing interest in preoperatively addressing modifiable patient comorbidity as a strategy to improve postoperative outcomes and reduce cost. Preoperative optimization can result in a quicker return to baseline functional capacity and has the potential to reduce postoperative complications.^11^ Although these effects are well-established in patients undergoing other abdominal operations,^12^ the benefits of preoperative risk reduction in patients undergoing VIHR are still unclear. A 2018 randomized clinical trial^13^ demonstrated that patients who were obese who underwent a focused weight loss program before VIHR had a modest reduction in 30-day postoperative complications. However, this trial only addressed obesity as a risk factor. It is unknown to what extent other preoperative risk factors may be targets for risk reduction. The financial burden of these complications and the potential savings that risk reduction may afford are also unclear, to our knowledge. Prior work has broadly characterized patients who may be at high risk while undergoing VIHR but has not clarified the extent to which individual risk factors contribute to poor outcomes or how modifying individual risk factors may improve clinical outcomes.^14^ Additionally, while risk factors have been shown to be a significant driver of surgical spending, little is known about the financial associations of individual comorbidities.^15^ Targeted and effective preoperative risk reduction prior to VIHR requires a better understanding of the most important preoperative risk factors, their association with postoperative morbidity, and the potential outcomes of risk reduction.

Within this context, we sought to identify the most significant modifiable comorbidities associated with complications after VIHR as well as their relative financial associations with total episode spending for surgical care. Importantly, we analyzed the individual associations of specific modifiable risk factors to better understand how targeted preoperative risk reduction may be associated with clinical outcomes and health care spending. This was conducted using a statewide clinical registry as well as a statewide multipayer claims registry. We hypothesize that specific modifiable risk factors are associated with postoperative complications and increased cost, which could facilitate the development of more effective preoperative optimization for VIHR.

## Methods

This study was approved by the institutional review board of the University of Michigan. The requirement for informed consent was waived because data were deidentified. This study followed the Strengthening the Reporting of Observational Studies in Epidemiology (STROBE) reporting guideline.

### Patient Population

Clinical data for this study were obtained from the Michigan Surgical Quality Collaborative (MSQC). The MSQC is a statewide quality improvement collaborative of 73 hospitals that share data and processes to improve surgical care and outcomes.^16^ The MSQC maintains a clinical registry of prospectively collected data on patient demographic characteristics, perioperative care processes, and 30-day outcomes after general surgical procedures in the state of Michigan. Hospitals that provide data to this registry receive funding from Blue Cross Blue Shield of Michigan to support trained nurses who perform data abstraction. Cases are reviewed using a sampling algorithm designed to minimize selection bias and represent 90% of eligible cases, or approximately 50 000 cases per year.^17^

Patients were included if they were adult patients (aged ≥18 years) who underwent elective VIHR from January 1, 2012, to December 31, 2018, including open and laparoscopic primary, incisional, and umbilical hernia repair. These procedures were identified using *Current Procedural Terminology* codes 49560, 49561, 49565, 49566, 49585, 49587, 49652, 49653, 49654, 49655, 49656, and 49657. Within this data set, each individual surgeon and hospital has a unique identifier, allowing for analysis of these factors as covariates.

### Primary Outcomes

Primary outcomes were complications after surgical procedures. Specifically, we chose to examine any complication, serious complications, discharge not to home, readmission within 30 days, and utilization of an emergency department (ED) within 30 days. The specific complications included in the categories any complications and serious complications are outlined in the eAppendix in the Supplement.

### Cost Estimation

Total 90-day episode spending was calculated for all primary outcomes. These data were collected from the Michigan Value Collaborative (MVC), which maintains a registry of administrative claims submitted directly to Blue Cross Blue Shield of Michigan, the state’s largest insurer, or Medicare fee-for-service. Episode spending in the MVC claims registry is price standardized and risk adjusted.^18^ The MVC claims registry has been validated and used previously to estimate the cost of care associated with complications and readmissions, and episode spending estimates are similar to those reported elsewhere.^19,20,21^

While episode spending data were derived from an overlapping but distinct patient data set, these values are intended to serve as representative examples of the relative financial burden of each complication. Although not directly linked to the patient cohort under analysis, financial data from the MVC cohort serves as a robust data set from which to generate point estimates of complication costs.

### Statistical Analysis

Our primary analysis was aimed at determining the relative contribution of various preoperative risk factors for complications after surgical procedures. All outcomes were binary. Given the hierarchically nested nature of data from hospitals and surgeons across the state, we used a multilevel mixed-effects logistic regression model to examine the contribution of patient-specific risk factors to each primary outcome while controlling for random effects at the surgeon and hospital levels. Patient-level fixed factors in each model were patient age, sex, self-reported race/ethnicity, American Society of Anesthesiologists (ASA) classification, body mass index (BMI; calculated as weight in kilograms divided by height in meters squared), nonindependence (defined as needing assistance from another person in performing ≥1 activity of daily living), hypertension, congestive heart failure, chronic obstructive pulmonary disease, coronary artery disease, peripheral vascular disease, bleeding disorder, deep venous thrombosis (DVT), sleep apnea, unhealthy alcohol use, smoking status, diabetes, open surgical technique, and inpatient surgical procedure.

Descriptive analyses of total episode spending for patients without any complication and with our outcomes of interest were performed using spending medians with an interquartile range (IQR). We estimated the added spending associated with an outcome as the difference between the median episode spending for patients with the outcome and patients without any complication. We multiplied the added spending from an outcome by the number of patients experiencing the outcome to get the cumulative spending associated with an outcome.

We estimated the attributable risk (AR) and population attributable risk fraction (PARF), which measure the contribution of modifiable risk factors on outcomes. The AR represents the proportion of the adverse outcomes that can be attributed to a modifiable risk factor among patients with that modifiable risk factor, and it was estimated using the adjusted odds ratio (aOR) for association of the modifiable risk factor with adverse outcome: AR = (OR − 1)/OR. The PARF represents the percentage of adverse outcomes that can be attributed to a modifiable risk factor across the entire population of patients undergoing VIHR, and it was estimated using the aOR for the modifiable risk factor and the percentage of the population exposed to the modifiable risk factor: PARF = [% exposed × (aOR − 1)]/[% exposed × aOR]. We calculated the mean and cumulative episode spending attributable to each risk factor through poor outcomes by multiplying PARF by the mean and cumulative added episode payment for an outcome, respectively. Statistical analyses were performed using Stata statistical software version 15.1 (StataCorp). *P* values were 2-tailed, and statistical significance was set at less than .05. Data were analyzed from April 2018 to September 2018.

## Results

Data were collected on 22 664 patients (median [IQR] age, 55 [44-64] years; 10 496 [46.3%] women) who met inclusion criteria across 73 hospitals and 896 surgeons. Full demographic data are listed in Table 1.

**Table 1.** Demographic and Procedural Characteristics of Cohort

Characteristic	No. (%)
Age, y	
<45	5945 (26.2)
45-64	11 191 (49.4)
≥65	5528 (24.4)
Women	10 496 (46.3)
Race	
White	19 252 (84.9)
Black	2452 (10.8)
Other	960 (4.2)
ASA classification	
I	1549 (6.8)
II	11 763 (51.9)
III	8871 (39.1)
IV	467 (2.1)
Body mass index quartile	
First	5737 (25.3)
Second	5844 (25.8)
Third	5703 (25.2)
Fourth	5343 (23.6)
Nonindependent risk factors	224 (1.0)
Hypertension	10 691 (47.2)
Congestive heart failure	57 (0.3)
Chronic obstructive pulmonary disease	1874 (8.3)
Coronary artery disease	2348 (10.4)
Peripheral vascular disease	450 (2.0)
Bleeding disorder	473 (2.1)
Deep vein thrombosis	1124 (5.0)
Obstructive sleep apnea	5754 (25.4)
Unhealthy alcohol use	657 (2.9)
Smoking	5492 (24.2)
Diabetes status	
None	18 472 (81.5)
Diet-controlled	605 (2.7)
Non–insulin-dependent	2487 (11.0)
Insulin-dependent	1100 (4.9)
Open surgical technique	15 868 (70.0)
Inpatient status	6974 (30.8)

Abbreviation: ASA, American Society of Anesthesiologists.

Mean (SD) BMI was 24.32 (2.56) in the first quartile, 29.72 (1.21) in the second quartile, 34.32 (1.51) in the third quartile, and 43.29 (6.18) in the fourth quartile. Most patients (18 472 patients [81.5%]) did not have diabetes, but 605 patients (2.7%) had diet-controlled diabetes, 2487 patients (11.0%) had medication-controlled but not insulin-dependent diabetes, and 1100 patients (4.9%) had insulin-dependent diabetes. Preoperative hemoglobin A_1c_ (HbA_1c_) was available for 2610 patients (12%). Mean (SD) HbA_1c_ was 5.8% (1.2%) of total hemoglobin (to convert to proportion of total hemoglobin, multiply by 0.01) for patients without diabetes, 6.2% (0.8%) of total hemoglobin for patients with diet-controlled diabetes, 6.9% (1.3%) of total hemoglobin for patients with oral medication-controlled diabetes, and 8.0% (1.8%) of total hemoglobin for patients with insulin-dependent diabetes (*P* < .001).

Most VIHRs (15 868 [70.0%]) were performed with an open surgical technique. Among patients who underwent open VIHR, 4275 patients (26.9%) were in the first quartile of BMI, 4249 patients (26.8%) were in the second quartile of BMI, 3952 patients (24.9%) were in the third quartile of BMI, and 3369 patients (21.2%) were in the fourth quartile of BMI. Most VIHRs (15 690 patients [69.2%]) were performed on an outpatient basis.

### Postoperative Complications

Within this cohort, 905 patients (4.0%) experienced any complication, 431 patients (1.9%) experienced a serious complication, 813 patients (3.6%) were discharged not to home, 1728 patients (7.6%) visited an ED within 30 days of surgery, and 922 patients (4.1%) were readmitted to the hospital within 30 days of surgery. After controlling for surgeon- and hospital-level variables, risk factors for any complication, serious complication, discharge not to home, 30-day ED utilization, and 30-day readmission are listed in Table 2.

**Table 2.** Multilevel Mixed-Effects Logistic Regression Model of Primary Outcomes

Characteristic	Any Complication		Serious Complication		Discharged Not to Home		30-d ED Utilization		30-d Readmission	
	aOR (95% CI)	*P* Value	aOR (95% CI)	*P* Value	aOR (95% CI)	*P* Value	aOR (95% CI)	*P* Value	aOR (95% CI)	*P* Value
Age, y										
<45	1 [Reference]		1 [Reference]		1 [Reference]		1 [Reference]		1 [Reference]	
45-64	1.00 (0.81-1.25)	.97	1.25 (0.91-1.73)	.17	2.21 (1.56-3.13)	<.001	0.55 (0.48-0.64)	<.001	0.95 (0.77-1.17)	.61
≥65	1.00 (0.77-1.29)	.98	1.12 (0.77-1.63)	.54	4.16 (2.88-6.01)	<.001	0.44 (0.37-0.53)	<.001	0.75 (0.59-0.97)	.03
Female sex	1.21 (1.03-1.41)	.02	1.21 (0.97-1.51)	.09	1.44 (1.20-1.72)	<.001	1.29 (1.15-1.44)	<.001	1.15 (0.98-1.34)	.08
Race/ethnicity										
White	1 [Reference]		1 [Reference]		1 [Reference]		1 [Reference]		1 [Reference]	
Black	0.90 (0.70-1.15)	.39	1.02 (0.74-1.40)	.91	1.04 (0.79-1.36)	.80	1.50 (1.27-1.76)	<.001	1.30 (1.04-1.62)	.02
Other	1.04 (0.70-1.53)	.85	1.00 (0.58-1.72)	>.99	0.84 (0.52-1.37)	.49	0.86 (0.64-1.16)	.31	0.91 (0.61-1.36)	.66
ASA classification										
I	1 [Reference]		1 [Reference]		1 [Reference]		1 [Reference]		1 [Reference]	
II	1.90 (1.02-3.54)	.04	1.38 (0.59-3.20)	.46	6.39 (0.88-46.55)	.07	1.54 (1.15-2.05)	.004	1.61 (0.96-2.71)	.07
III	2.63 (1.40-4.96)	.003	1.95 (0.83-4.59)	.13	11.48 (1.57-83.72)	.02	1.99 (1.46-2.71)	<.001	2.41 (1.41-4.12)	.001
IV	3.47 (1.69-7.13)	.001	3.15 (1.21-8.17)	.02	17.38 (2.31-130.73)	.006	2.59 (1.67-4.02)	<.001	3.15 (1.68-5.92)	<.001
BMI quartile										
First	1 [Reference]		1 [Reference]		1 [Reference]		1 [Reference]		1 [Reference]	
Second	1.15 (0.91-1.45)	.23	1.11 (0.80-1.54)	.54	1.06 (0.83-1.36)	.63	0.92 (0.78-1.08)	.32	0.91 (0.74-1.14)	.42
Third	1.22 (0.97-1.54)	.09	1.21 (0.88-1.68)	.24	0.95 (0.73-1.2)	.68	1.01 (0.86-1.18)	.92	1.01 (0.82-1.25)	.93
Fourth	1.64 (1.30-2.06)	<.001	1.67 (1.22-2.31)	.002	1.07 (0.83-1.39)	.60	1.04 (0.88-1.23)	.62	0.96 (0.77-1.20)	.75
Nonindependent characteristics	1.53 (0.99-2.38)	.06	1.61 (0.93-2.78)	.09	6.21 (4.24-9.09)	<.001	0.61 (0.35-1.07)	.08	1.49 (0.96-2.32)	.08
Hypertension	1.08 (0.91-1.28)	.40	1.08 (0.84-1.37)	.56	1.09 (0.89-1.33)	.40	1.00 (0.88-1.14)	.95	1.12 (0.94-1.32)	.21
Congestive heart failure	1.43 (0.58-0.35)	.43	1.04 (0.30-3.63)	.95	3.12 (1.29-7.55)	.01	0.65 (0.22-1.90)	.44	0.92 (0.34-2.50)	.88
Chronic obstructive pulmonary disease	1.30 (1.05-1.62)	.02	1.22 (0.91-1.64)	.18	1.25 (0.98-1.58)	.07	1.07 (0.87-1.28)	.50	1.13 (0.91-1.41)	.27
Coronary artery disease	1.26 (1.02-1.55)	.03	1.13 (0.84-1.50)	.42	1.00 (0.80-1.25)	.99	1.25 (1.04-1.50)	.02	1.44 (1.17-1.76)	<.001
Peripheral vascular disease	1.24 (0.86-1.78)	.26	1.38 (0.86-2.20)	.19	1.29 (0.88-1.89)	.19	1.37 (1.01-1.88)	.046	1.10 (0.76-1.59)	.61
Bleeding disorder	1.37 (0.96-1.95)	.08	1.24 (0.77-2.00)	.37	0.91 (0.61-1.37)	.66	1.05 (0.75-1.47)	.77	1.36 (0.96-1.91)	.08
Deep vein thrombosis	1.95 (1.57-2.42)	<.001	1.93 (1.45-2.57)	<.001	1.66 (1.31-2.11)	<.001	1.58 (1.29-1.92)	<.001	1.86 (1.50-2.32)	<.001
Obstructive sleep apnea	1.14 (0.96-1.36)	.13	1.01 (0.80-1.28)	.94	1.05 (0.86-1.27)	.63	1.13 (0.99-1.29)	.08	1.13 (0.95-1.34)	.16
Unhealthy alcohol use	1.42 (0.92-2.19)	.11	1.75 (1.00-3.07)	.049	1.09 (0.60-1.99)	.78	1.18 (0.86-1.62)	.31	1.11 (0.70-1.74)	.66
Smoking	1.01 (0.85.-1.22)	.87	1.05 (0.82-1.35	.70	1.16 (0.93-1.43)	.18	1.33 (1.17-1.50)	<.001	0.98 (0.82-1.61)	.78
Diabetes status										
None	1 [Reference]		1 [Reference]		1 [Reference]		1 [Reference]		1 [Reference]	
Diet-controlled	0.96 (0.65-1.41)	.83	0.96 (0.56-1.63)	.87	1.31 (0.91-1.90)	.15	1.27 (0.84-1.70)	.12	0.79 (0.52-1.20)	.28
Non–insulin-dependent	0.97 (0.78-1.21)	.79	1.19 (0.87-1.59)	.25	1.26 (1.00-1.59)	.05	1.03 (0.86-1.22)	.78	1.10 (0.83-1.28)	.79
Insulin-dependent	1.34 (1.03-1.73)	.03	1.51 (1.08-2.12)	.02	1.49 (1.12-1.98)	.005	1.23 (0.98-1.54)	.08	1.68 (1.32-2.14)	<.001
Open surgical technique	2.43 (2.01-2.95)	<.001	2.09 (1.62-2.70)	<.001	2.43 (1.95-3.03)	<.001	1.00 (0.88-1.13)	.96	1.19 (1.01-1.41)	.04
Inpatient status	4.30 (3.60-5.12)	<.001	5.58 (4.30-7.25)	<.001	34.37 (23.18-50.96)	<.001	1.28 (1.13-1.44)	<.001	3.09 (2.62-3.63)	<.001

Abbreviations: aOR, adjusted odds ratio; ASA, American Society of Anesthesiologists; ED, emergency department.

Common significant risk factors for any complications and serious complications included having fourth-quartile BMI (any: aOR, 1.64; 95% CI, 1.30-2.06; *P* < .001; serious: aOR, 1.67; 95% CI, 1.22-2.31; *P* = .002), ASA class IV (any: aOR, 3.47; 95% CI, 1.69-7.13; *P* = .001; serious: aOR, 3.15; 95% CI, 1.21-8.17; *P* = .02), DVT (any: aOR, 1.95; 95% CI, 1.57-2.42; *P* < .001; serious: aOR, 1.93; 95% CI, 1.45-2.57; *P* < .001), insulin-dependent diabetes (any: aOR, 1.34; 95% CI, 1.03-1.73; *P* = .03; serious: aOR, 1.51; 95% CI, 1.08-2.12; *P* = .02), open surgical technique (any: aOR, 2.43; 95% CI, 2.01-2.95; *P* < .001; serious: aOR, 2.09; 95% CI, 1.62-2.70; *P* < .001), and inpatient status (any: aOR, 4.30; 95% CI, 3.60-5.12; *P* < .001; serious: aOR, 5.58; 95% CI, 4.30-7.25; *P* < .001).

Significant risk factors for discharge not to home were age 45 to 64 years (aOR, 2.21; 95% CI, 1.56-3.13; *P* < .001) or 65 years or older (aOR, 4.16; 95% CI, 2.88-6.01; *P* < .001), female sex (aOR, 1.44; 95% CI, 1.20-1.72; *P* < .001), ASA class III (aOR, 11.48; 95% CI, 1.57-83.72; *P* = .02) or class IV (aOR, 17.38; 95% CI, 2.31-130.73; *P* = .006), nonindependent functional status (aOR, 6.21; 95% CI, 4.24-9.09; *P* < .001), congestive heart failure (aOR, 3.12; 95% CI, 1.29-7.55; *P* = .01), DVT (aOR, 1.66; 95% CI, 1.31-2.11; *P* < .001), insulin-dependent diabetes (aOR, 1.49; 95% CI, 1.12-1.98; *P* = .005), open surgical technique (aOR, 2.43; 95% CI, 1.95-3.03; *P* < .001), and inpatient status (aOR, 34.37; 95% CI, 23.18-50.96; *P* < .001).

Significant risk factors for 30-day ED utilization were female sex (aOR, 1.29; 95% CI, 1.15-1.44; *P* < .001), black race (aOR, 1.50; 95% CI, 1.27-1.76; *P* < .001), ASA class II (aOR, 1.54; 95% CI, 1.15-2.05; *P* = .004), ASA class III (aOR, 1.99; 95% CI, 1.46-2.71; *P* < .001), ASA class IV (aOR, 2.59; 95% CI, 1.67-4.02; *P* < .001), coronary artery disease (aOR, 1.25; 95% CI, 1.04-1.50; *P* = .02), peripheral vascular disease (aOR, 1.37; 95% CI, 1.01-1.88; *P* = .046), DVT (aOR, 1.58; 95% CI, 1.229-1.92; *P* < .001), smoking (aOR, 1.33; 95% CI, 1.17-1.50; *P* < .001), and inpatient status (aOR, 1.28; 95% CI, 1.13-1.44; *P* < .001). Older patients were significantly less likely to visit an ED within 30 days of VIHR (age 45-64 years: aOR, 0.55; 95% CI, 0.48-0.64; *P* < .001; age ≥65 years: aOR, 0.44; 95% CI, 0.37-0.53; *P* < .001).

Additionally, significant risk factors for 30-day hospital readmission were black race (aOR, 1.30; 95% CI, 1.04-1.62; *P* = .02), ASA class III (aOR, 2.41; 95% CI, 1.41-4.12; *P* = .001) or class IV (aOR, 3.15; 95% CI, 1.68-5.92; *P* < .001), coronary artery disease (aOR, 1.44; 95% CI, 1.17-1.76; *P* < .001), DVT (aOR, 1.86; 95% CI, 1.50-2.32; *P* < .001), insulin-dependent diabetes (aOR, 1.68; 95% CI, 1.32-2.14; *P* < .001), open surgical technique (aOR, 1.19; 95% CI, 1.01-1.41; *P* = .04), and inpatient status (aOR, 3.09; 95% CI, 2.62-3.63; *P* < .001). Being 65 years or older was associated with reduced odds of 30-day hospital readmission (aOR, 0.75; 95% CI, 0.59-0.97; *P* = .03).

### Ninety-Day Episode Spending

Total episode spending was available for a similar cohort of 24 159 patients undergoing VIHR in Michigan (Table 3). Among these patients, 19 360 patients (80.1%) did not experience any postoperative complications, and median (IQR) total episode payment was $6905 ($4520-$9851). The median (IQR) total episode payment was $16 839 ($13 744-$21 702) for patients with any complication, $33 553 ($25 152-$43 017) for patients with serious complication, $23 872 ($15 974-$36 079) for patients with discharge not to home, $11 918 ($7867-$19 342) for patients with 30-day ED utilization, and $25 775 ($18 282-$38 536) for patients with 30-day readmission.

**Table 3.** Spending Associated With Each Outcome

Outcome	No. (%)	Episode Spending, Median (IQR), $		Added Spending Per Episode, Median (IQR) [% of Total], $
		Estimate	Total From Outcome	
No complication	19 360 (85.4)	6905 (4520-9851)	0	0
Any complication	905 (4.0)	16 839 (13 744-21 702)	8 990 270 (8 347 720-10 725 155)	9934 (9224-11 851) [59]
Serious complication	431 (1.9)	33 553 (25 152-43 017)	11 485 288 (8 892 392-14 294 546)	26 648 (20 632-33 166) [79]
Discharge not to home	813 (3.6)	23 872 (15 974-36 079)	13 794 171 (9 312 102-21 323 364)	16 967 (11 454-26 228) [71]
30-d ED utilization	1728 (7.6)	11 918 (7867-19 342)	8 662 464 (5 783 616-16 400 448)	5013 (3347-9491) [42]
30-d readmission	922 (4.1)	25 775 (18 282-38 536)	17 398 140 (12 688 564-26 447 570)	18 870 (13 762-28 685) [73]
Total spending			60 330 333 (45 024 394-89 191 083)	

Abbreviations: ED, emergency department; IQR, interquartile range.

Additional episode spending for an outcome ranged from a median (IQR) of $5013 ($3347-$9491) for 30-day ED utilization to $26 648 ($20 632-$33 166) for serious complication. Median (IQR) cumulative episode spending for this cohort ranged from $8 662 464 ($5 783 616-$16 400 448) for 30-day ED utilization to $17 398 140 ($12 688 564-$26 447 570) for 30-day readmission. Median (IQR) cumulative additional spending from these complications for the entire cohort was $60 330 333 ($45 024 394-$89 191 083).

To quantify the financial outcome associated with specific modifiable preoperative comorbidities, AR was calculated for modifiable risk factors that significantly increased the odds of postoperative complications, including fourth-quartile BMI, unhealthy alcohol use, smoking, and insulin-dependent diabetes (Table 4). For example, among patients with the risk factor of being in the fourth-quartile of BMI, 39.0% (AR = 0.390) of complications were attributed to this risk factor. Assuming 23.6% of the population had fourth-quartile BMI, the PARF of all adverse outcomes in all patients undergoing VIHR that could be attributed to fourth-quartile BMI is 13.1%. Similarly, among patients with the risk factor of insulin-dependent diabetes, 40.5% (AR = 0.405) of 30-day readmissions were attributable to this risk factor. Assuming 4.9% of the population had insulin-dependent diabetes, the PARF of all 30-day readmissions in all patients undergoing VIHR that would be attributable to this risk factor is 3.2%. The additional spending attributable to these factors is presented in Table 4. There was a range of additional spending depending on risk factor and outcome. Fourth-quartile BMI was associated with the largest amount of additional spending. For patients who experienced a serious complication, fourth-quartile BMI accounted for $3638 (PARF = 13.7%) of additional spending per patient, and a cumulative additional spending of $1 568 105. Fourth-quartile BMI also accounted for $1304 (PARF = 13.1%) of per-patient additional spending for patients who experienced any complication, amounting to a cumulative $1 179 707 in additional spending. This is compared to unhealthy alcohol use, which accounted for $567 (PARF = 2.1%) of additional spending per patient for serious complication, and insulin-dependent diabetes, which accounted for an additional $650 (PARF = 2.4%) of additional spending per patient for serious complications. Smoking accounted for $371 (PARF = 7.4%) of additional spending per patient for 30-day ED utilization. Insulin-dependent diabetes also accounted for an additional per patient spending of $163 (PARF = 1.6%) for any complication, $398 (PARF = 2.3%) for discharge not to home, and $608 (PARF = 3.2%) for 30-day readmission.

**Table 4.** Added Episode and Cumulative Spending Attributed to Significant Modifiable Preoperative Risk Factors

Risk Factor	Patients With Exposure, %	AR	PARF, %	Added Spending Attributed to Risk Factor, $	
				Per Episode, Median (IQR) [% of Additional Spending]^a^	Cumulative, Median (IQR)^a^
Fourth-quartile BMI					
Any complication	23.6	0.390	13.1	1301 (1208-1552) [13]	1 177 725 (1 093 551-1 404 995)
Serious complication	23.6	0.401	13.7	3651 (2827-4544) [14]	1 573 484 (1 218 258-1 958 353)
Unhealthy alcohol use					
Serious complication	2.9	0.429	2.1	560 (433-696) [2]	241 191 (186 740-300 185)
Smoking					
30-d ED utilization	24.2	0.248	7.4	371 (248-702) [7]	641 022 (427 988-1 213 633)
Insulin dependence					
Any complication	4.9	0.254	1.6	159 (148-190) [2]	143 844 (133 564-171 602)
Serious complication	4.9	0.338	2.4	640 (495-796) [2]	275 647 (213 417-343 069)
Discharge not to home	4.9	0.329	2.3	390 (263-603) [2]	317 266 (214 178-490 437)
30-d readmission	4.9	0.405	3.2	604 (440-918) [3]	556 740 (406 034-846 322)

Abbreviations: AR, attributable risk; BMI, body mass index; ED, emergency department; IQR, interquartile range; PARF, population attributable risk fraction.

^a^ Calculated as PARF × Added Spending from Outcome.

## Discussion

This cross-sectional study found several significant risk factors associated with postoperative outcomes for patients undergoing VIHR after controlling for hospital- and surgeon-level variation. These complications were associated with approximately $5000 to $27 000 in increased spending per episode of care, depending on outcome. Given the ubiquity of VIHR, this increased spending represents a significant financial burden to the health care system. In our regional cohort of patients undergoing VIHR in Michigan, the spending associated with postoperative complications was estimated to exceed $90 million for more than 20 000 patients in a 4-year period. Although some of the risk factors identified in this study are fixed, other common risk factors, such as morbid obesity, insulin-dependent diabetes, unhealthy alcohol use, and smoking, are potentially modifiable prior to undergoing a surgical procedure. While these risk factors have previously been analyzed in similar populations, this is the first study to quantify the AR and financial burden of modifiable risk factors in patients undergoing VIHR, to our knowledge. This quantifiable measurement of the clinical and financial effect of these factors could help surgeons with finite resources to strategically deploy and implement quality improvement initiatives in the areas in which they may have the greatest impact.

It has been previously demonstrated that preoperative optimization, or prehabilitation, has the potential to reduce length of stay, improve postoperative outcomes, and accelerate return to baseline functional status.^12,22,23^ These programs typically involve efforts directed at improving activity level (eg, ambulation, incentive spirometry).^24^ Comorbidities are common in patients who develop ventral or incisional hernias, so preoperative optimization has the potential to impart significant improvement in outcomes. However, development of effective preoperative optimization for patients undergoing VIHR requires knowledge of which comorbidities may contribute to postoperative complications.

This study identifies a number of fixed and modifiable risk factors. Specifically, obesity in the fourth quartile (compared with the first quartile) of our cohort was a risk factor for any complications and serious complications after surgical procedures, and insulin-dependent diabetes was a risk factor for any complication, serious complication, discharge not to home, and 30-day readmission. Importantly, fourth-quartile BMI (compared with the first quartile) was associated with the most additional spending per complication. For example, it accounted for an additional $3638 spending on serious complications, while unhealthy alcohol use accounted for an additional $567 spent on serious complications, and insulin-dependent diabetes accounted for an additional $650 spent on serious complications. Importantly, nearly half of patients undergoing VIHR are obese,^25^ and more than one-quarter of patients in our cohort had a BMI greater than 40. This presents a significant opportunity for improvement in spending.^26^ Weight loss prior to undergoing surgical procedures has already been demonstrated to benefit patients undergoing VIHR.^13^ Patients with diabetes and who have poor preoperative glucose control are also at higher risk of morbidity when undergoing other operations.^27,28^ Additionally, unhealthy alcohol use was shown to be a risk factor for serious complication after surgical procedures, and smoking was a risk factor for 30-day ED utilization. Although smoking was a significant risk factor for only 1 outcome, it is well established that smoking is one of the most significant risk factors for poor outcomes after VIHR.^29,30^ This likely reflects widespread patient selection, whereby smokers more commonly undergo watchful waiting or are offered a minimally invasive approach, which is associated with fewer complications. Similarly in our cohort, the association of open surgical technique with increased complications likely reflects patient selection, wherein patient- and hernia-related factors (ie, comorbidities, hernia size) preclude the use of a minimally invasive approach.

Therefore, our analysis suggests that effective prehabilitation should at minimum involve weight loss, particularly for patients whose BMI exceeds 40, and glycemic control, particularly for patients whose HbA_1c_ level exceeds 8.0% of total hemoglobin. Preoperative weight loss interventions may include patient participation in a structured exercise and diet program, as well as referral to a bariatric surgeon when appropriate.^4^ Patients whose HbA_1c_ level is greater than 8.0% of total hemoglobin should work with an endocrinologist to achieve better glucose control prior to proceeding with surgery. Previous studies have found that an HbA_1c_ level greater than 6% to 7% of total hemoglobin increased the risk of postoperative complications by an OR of 1.7 to 5.8, and a 2017 consensus recommended avoiding elective surgical procedures in patients whose HbA_1c_ level exceeded 8.0% of total hemoglobin.^7,31,32^ While the risk of emergency surgical procedures is relatively low, it is not 0.^9^ Moreover, nonoperative management results in poor quality of life and functional status.^10,33^ In a 2016 prospective, case-matched study^10^ of operative vs nonoperative management of ventral hernia, operative management improved overall function and quality of life. Another prospective study from 2019^34^ that compared long-term outcomes of operative vs nonoperative management found that this improvement was sustained 3 years after the surgical procedure and that nearly 40% of patients in the nonoperative group ultimately went on to undergo hernia repair. A targeted prehabilitation program for VIHR has the potential to transform these patients into operative candidates.

It is important to note that some key comorbidities identified in this study, such as obesity, diabetes, and unhealthy alcohol use, can be difficult to modify. Weight regain after medically supervised weight loss is very common, raising the question of whether patients who do lose weight prior to VIHR will be able to maintain their target weight.^35^ A study by Rosen et al^36^ demonstrated that a multidisciplinary preoperative weight loss program involving a protein-sparing fast prior to abdominal wall reconstruction could achieve 37% reduction in excess BMI, which was maintained at 18 months postoperatively. Moreover, there is high-quality evidence from a randomized clinical trial from 2018^13^ demonstrating that preoperative nutritional counseling and exercise were associated with weight loss and lower complication rate. Improving perioperative glycemic control has also been demonstrated to improve surgical outcomes.^37,38,39^ Given the well-established association of obesity with diabetes, a durable solution may be found in preoperative bariatric surgical procedures, which have been shown to achieve not only lasting weight loss, but diabetes resolution as well.^21^ Nevertheless, these goals are still difficult to achieve, highlighting the importance of patient selection.

These data also suggest that targeting high-risk patients represents an opportunity to substantially reduce health care expenditures. Although it has been previously demonstrated that medical reimbursement is significantly higher at hospitals with high rates of complications, these data reflect the extent to which these complications are specifically associated with patient-level factors.^40^ Median cumulative additional spending in this cohort was approximately $60 million. Based on these data, a 25% reduction in serious complications after VIHR could result in a median savings of roughly $3.6 million. Similarly, a 25% decrease in 30-day readmissions could result in a median savings of nearly $6 million. Furthermore, cumulative additional spending specifically associated with fourth-quartile BMI, unhealthy alcohol use, smoking, and insulin-dependent diabetes totaled approximately $5 million. Preoperative optimization has been shown to confer modest savings to patients undergoing abdominal surgical procedures; however, definitive data are lacking regarding reduction in health care spending. These data suggest substantial savings are possible with preoperative optimization of select patients prior to VIHR. Additionally, these data support the use of outpatient-based minimally invasive surgical procedures. Preoperative risk reduction may then further improve outcomes by transitioning a patient who may have previously required admission owing to multiple comorbidities to one who can undergo a surgical procedure on an outpatient basis.^19^

### Limitations

This study had limitations, the first of which is the retrospective nature of these data. This introduces the possibility of unmeasured bias, especially in the form of patient selection. Risk-adjustment was used to mitigate potential confounding. An additional limitation is that this study examined only 30-day complication rates and 90-day episode-of-care spending. It is possible that long-term outcomes, such as hernia recurrence, may not be associated with preoperative risk reduction, given the high incidence of weight regain and the long-term metabolic effects of obesity, diabetes, and unhealthy alcohol use. Another limitation is that financial data, although obtained from an overlapping cohort of patients across Michigan, was not linked with individual patients. Our goal was to derive point estimates of episode spending that are representative of common complications for patients undergoing the same operation. These spending estimates may be significantly different in different populations or health systems. Moreover, risk factors were analyzed on an individual bases, and the cumulative risk of patients with multiple risk factors was not analyzed in this study. Therefore, it is likely that our estimates of spending and savings potential are conservative estimates, since many of the comorbidities under analysis occur simultaneously in many patients. This cohort also included a wide spectrum of hernias, from simple umbilical hernias to complex incisional hernias requiring open reconstruction. The heterogeneity of this group, while representative of the population, limits conclusions about risk modification for patients at each end of the spectrum. For example, preoperative risk reduction may only play a marginal role for patients undergoing simple umbilical hernia repair. While this study sought to identify broad clinical associations and health care spending at a population level, future work is needed to understand the nuanced differences between patients undergoing routine hernia repair vs patients who are high risk and who undergo complex abdominal wall reconstruction, in whom preoperative optimization may play an even more significant role. Although hernia size was unknown in this population-level cohort, this analysis nevertheless gives providers modifiable goals. Our study controlled for hernia complexity, which is related to size, by controlling for surrogate factors, such as minimally invasive vs open repair, as well as inpatient status. Additionally, we used aORs to estimate risk reductions, which may overestimate the effect of certain comorbidities on adverse outcomes. However, when event rates are low, as was the case in our study, the aOR can approximate relative risk.

## Conclusions

This study found that, after controlling for surgeon- and hospital-level variation, a number of adverse postoperative outcomes and increased spending were associated with patient-level preoperative risk factors. A number of these factors, such as obesity, diabetes, unhealthy alcohol use, and smoking, are potentially modifiable. Preoperatively modifying these risk factors may therefore improve patient outcomes and reduce the health care expenditures associated with these complications. This specific understanding of the association of specific comorbidities with postoperative recovery could allow for the development of a targeted program of preoperative optimization, which is already the subject of quality improvement work being conducted on a statewide basis.

## Notes

Supplement. **eAppendix.** Definition of Complications

## References

[1] Poulose BK, Shelton J, Phillips S, Epidemiology and cost of ventral hernia repair: making the case for hernia research. Hernia. 2012;16(2):-. doi:10.1007/s10029-011-0879-9 21904861

[2] Luijendijk RW, Hop WC, van den Tol MP, A comparison of suture repair with mesh repair for incisional hernia. N Engl J Med. 2000;343(6):392-398. doi:10.1056/NEJM200008103430603 10933738

[3] Burger JW, Luijendijk RW, Hop WC, Halm JA, Verdaasdonk EG, Jeekel J Long-term follow-up of a randomized controlled trial of suture versus mesh repair of incisional hernia. Ann Surg. 2004;240(4):578-583. doi:10.1097/01.sla.0000141193.08524.e7 15383785 PMC1356459

[4] Liang MK, Holihan JL, Itani K, Ventral hernia management: expert consensus guided by systematic review. Ann Surg. 2017;265(1):80-89. doi:10.1097/SLA.0000000000001701 28009730

[5] Basta MN, Fischer JP, Wink JD, Kovach SJ Mortality after inpatient open ventral hernia repair: developing a risk stratification tool based on 55,760 operations. Am J Surg. 2016;211(6):1047-1057. doi:10.1016/j.amjsurg.2015.03.009 26975662

[6] Lindmark M, Strigård K, Löwenmark T, Dahlstrand U, Gunnarsson U Risk factors for surgical complications in ventral hernia repair. World J Surg. 2018;42(11):3528-3536. doi:10.1007/s00268-018-4642-6 29700567 PMC6182761

[7] Goodenough CJ, Ko TC, Kao LS, Development and validation of a risk stratification score for ventral incisional hernia after abdominal surgery: hernia expectation rates in intra-abdominal surgery (the HERNIA Project). J Am Coll Surg. 2015;220(4):405-413. doi:10.1016/j.jamcollsurg.2014.12.027 25690673 PMC4372474

[8] Cox TC, Blair LJ, Huntington CR, The cost of preventable comorbidities on wound complications in open ventral hernia repair. J Surg Res. 2016;206(1):214-222. doi:10.1016/j.jss.2016.08.009 27916364

[9] Bellows CF, Robinson C, Fitzgibbons RJ, Webber LS, Berger DH Watchful waiting for ventral hernias: a longitudinal study. Am Surg. 2014;80(3):245-252. 24666865

[10] Holihan JL, Henchcliffe BE, Mo J, Is nonoperative management warranted in ventral hernia patients with comorbidities: a case-matched, prospective, patient-centered study. Ann Surg. 2016;264(4):585-590. doi:10.1097/SLA.0000000000001865 27355269

[11] Moran J, Guinan E, McCormick P, The ability of prehabilitation to influence postoperative outcome after intra-abdominal operation: a systematic review and meta-analysis. Surgery. 2016;160(5):1189-1201. doi:10.1016/j.surg.2016.05.014 27397681

[12] Howard R, Yin YS, McCandless L, Wang S, Englesbe M, Machado-Aranda D Taking control of your surgery: impact of a prehabilitation program on major abdominal surgery. J Am Coll Surg. 2019;228(1):72-80. doi:10.1016/j.jamcollsurg.2018.09.018 30359831 PMC6309718

[13] Liang MK, Bernardi K, Holihan JL, Modifying risks in ventral hernia patients with prehabilitation: a randomized controlled trial. Ann Surg. 2018;268(4):674-680. doi:10.1097/SLA.0000000000002961 30048306

[14] Lovecchio F, Farmer R, Souza J, Khavanin N, Dumanian GA, Kim JY Risk factors for 30-day readmission in patients undergoing ventral hernia repair. Surgery. 2014;155(4):702-710. doi:10.1016/j.surg.2013.12.021 24612622

[15] Davenport DL, Henderson WG, Khuri SF, Mentzer RM Jr Preoperative risk factors and surgical complexity are more predictive of costs than postoperative complications: a case study using the National Surgical Quality Improvement Program (NSQIP) database. Ann Surg. 2005;242(4):463-468. doi:10.1097/01.sla.0000183348.15117.ab 16192806 PMC1402344

[16] Campbell DA Jr, Dellinger EP Multihospital collaborations for surgical quality improvement. JAMA. 2009;302(14):1584-1585. doi:10.1001/jama.2009.1474 19826030

[17] Healy MA, Regenbogen SE, Kanters AE, Surgeon variation in complications with minimally invasive and open colectomy: results from the Michigan Surgical Quality Collaborative. JAMA Surg. 2017;152(9):860-867. doi:10.1001/jamasurg.2017.1527 28614551 PMC5710462

[18] Cleary RK, Mullard AJ, Ferraro J, Regenbogen SE The cost of conversion in robotic and laparoscopic colorectal surgery. Surg Endosc. 2018;32(3):1515-1524. doi:10.1007/s00464-017-5839-8 28916895 PMC7795723

[19] Sheetz KH, Kenney B, Dupree JM, Campbell DA, Englesbe MJ Targeting value-driven quality improvement for laparoscopic cholecystectomy in Michigan. Ann Surg. 2019;269(1):127-132. doi:10.1097/SLA.0000000000002438 28742681

[20] Ellimoottil C, Syrjamaki JD, Voit B, Guduguntla V, Miller DC, Dupree JM Validation of a claims-based algorithm to characterize episodes of care. Am J Manag Care. 2017;23(11):e382-e386. 29182359

[21] Aminian A, Zajichek A, Arterburn DE, Association of metabolic surgery with major adverse cardiovascular outcomes in patients with type 2 diabetes and obesity. JAMA. 2019. doi:10.1001/jama.2019.14231 31475297 PMC6724187

[22] Barberan-Garcia A, Ubré M, Roca J, Personalised prehabilitation in high-risk patients undergoing elective major abdominal surgery: a randomized blinded controlled trial. Ann Surg. 2018;267(1):50-56. doi:10.1097/SLA.0000000000002293 28489682

[23] Li C, Carli F, Lee L, Impact of a trimodal prehabilitation program on functional recovery after colorectal cancer surgery: a pilot study. Surg Endosc. 2013;27(4):1072-1082. doi:10.1007/s00464-012-2560-5 23052535

[24] Englesbe MJ, Grenda DR, Sullivan JA, The Michigan Surgical Home and Optimization Program is a scalable model to improve care and reduce costs. Surgery. 2017;161(6):1659-1666. doi:10.1016/j.surg.2016.12.021 28174000

[25] Berger RL, Li LT, Hicks SC, Davila JA, Kao LS, Liang MK Development and validation of a risk-stratification score for surgical site occurrence and surgical site infection after open ventral hernia repair. J Am Coll Surg. 2013;217(6):974-982. doi:10.1016/j.jamcollsurg.2013.08.003 24051068

[26] Fischer JP, Basta MN, Mirzabeigi MN, A risk model and cost analysis of incisional hernia after elective, abdominal surgery based upon 12,373 cases: the case for targeted prophylactic intervention. Ann Surg. 2016;263(5):1010-1017. doi:10.1097/SLA.0000000000001394 26465784 PMC4825112

[27] Duggan EW, Carlson K, Umpierrez GE Perioperative hyperglycemia management: an update. Anesthesiology. 2017;126(3):547-560. doi:10.1097/ALN.0000000000001515 28121636 PMC5309204

[28] Dronge AS, Perkal MF, Kancir S, Concato J, Aslan M, Rosenthal RA Long-term glycemic control and postoperative infectious complications. Arch Surg. 2006;141(4):375-380. doi:10.1001/archsurg.141.4.375 16618895

[29] Sørensen LT, Hemmingsen UB, Kirkeby LT, Kallehave F, Jørgensen LN Smoking is a risk factor for incisional hernia. Arch Surg. 2005;140(2):119-123. doi:10.1001/archsurg.140.2.119 15723991

[30] Borad NP, Merchant AM The effect of smoking on surgical outcomes in ventral hernia repair: a propensity score matched analysis of the National Surgical Quality Improvement Program data. Hernia. 2017;21(6):855-867. doi:10.1007/s10029-017-1664-1 28864961

[31] Kokotovic D, Sjølander H, Gögenur I, Helgstrand F Watchful waiting as a treatment strategy for patients with a ventral hernia appears to be safe. Hernia. 2016;20(2):281-287. doi:10.1007/s10029-016-1464-z 26838293

[32] Stenberg E, Szabo E, Näslund I; Scandinavian Obesity Surgery Registry Study Group Is glycosylated hemoglobin A1 c associated with increased risk for severe early postoperative complications in nondiabetics after laparoscopic gastric bypass? Surg Obes Relat Dis. 2014;10(5):801-805. doi:10.1016/j.soard.2014.05.005 25304835

[33] Holihan JL, Flores-Gonzalez JR, Mo J, Ko TC, Kao LS, Liang MK A prospective assessment of clinical and patient-reported outcomes of initial non-operative management of ventral hernias. World J Surg. 2017;41(5):1267-1273. doi:10.1007/s00268-016-3859-5 28050665

[34] Bernardi K, Martin AC, Holihan JL, Is non-operative management warranted in ventral hernia patients with comorbidities: a case-matched, prospective 3 year follow-up, patient-centered study. Am J Surg. 2019;S0002-9610(19)30416-7. doi:10.1016/j.amjsurg.2019.07.044 31421893

[35] Blomain ES, Dirhan DA, Valentino MA, Kim GW, Waldman SA Mechanisms of weight regain following weight loss. ISRN Obes. 2013;2013:210524. doi:10.1155/2013/210524 24533218 PMC3901982

[36] Rosen MJ, Aydogdu K, Grafmiller K, Petro CC, Faiman GH, Prabhu A A multidisciplinary approach to medical weight loss prior to complex abdominal wall reconstruction: is it feasible? J Gastrointest Surg. 2015;19(8):1399-1406. doi:10.1007/s11605-015-2856-6 26001369

[37] Joslyn NA, Esmonde NO, Martindale RG, Hansen J, Khansa I, Janis JE Evidence-based strategies for the prehabilitation of the abdominal wall reconstruction patient. Plast Reconstr Surg. 2018;142(3)(suppl):21S-29S. doi:10.1097/PRS.0000000000004835 30138261

[38] Endara M, Masden D, Goldstein J, Gondek S, Steinberg J, Attinger C The role of chronic and perioperative glucose management in high-risk surgical closures: a case for tighter glycemic control. Plast Reconstr Surg. 2013;132(4):996-1004. doi:10.1097/PRS.0b013e31829fe119 23783058

[39] Smith MD, McCall J, Plank L, Herbison GP, Soop M, Nygren J Preoperative carbohydrate treatment for enhancing recovery after elective surgery. Cochrane Database Syst Rev. 2014;(8):CD009161. doi:10.1002/14651858.CD009161.pub2 25121931 PMC11060647

[40] Birkmeyer JD, Gust C, Dimick JB, Birkmeyer NJ, Skinner JS Hospital quality and the cost of inpatient surgery in the United States. Ann Surg. 2012;255(1):1-5. doi:10.1097/SLA.0b013e3182402c17 22156928 PMC3249383

