# Supplementary material for: Costs Associated With Modifiable Risk Factors in Ventral and Incisional Hernia Repair
Source: JAMA Netw Open. 2019 Nov 27;2(11):e1916330. doi: 10.1001/jamanetworkopen.2019.16330 (PMC6902835; doi:10.1001/jamanetworkopen.2019.16330)
Supplement: Supplement. — eAppendix. Definition of Complications [file jamanetwopen-2-e1916330-s001.pdf]

Supplementary Online Content

Howard R, Thompson M, Fan Z, Englesbe M, Dimick JB, Telem DA. Costs associated with modifiable risk factors in ventral and incisional hernia repair. *JAMA Netw Open.* 2019;2(11):e1916330. doi:10.1001/jamanetworkopen.2019.16330

eAppendix. Definition of Complications

This supplementary material has been provided by the authors to give readers additional information about their work.

© 2019 Howard R et al. *JAMA Network Open.*

eAppendix. Definition of Complications

Any Complication

- Acute Kidney Injury
- Anastomotic leak (All)
- Cardiac Arrest req. CPR – Intraop
- Cardiac Arrest req. CPT - Postop
- C-difficile
- Deep Incisional SSI
- Deep Vein Thrombosis req. Therapy
- Myocardial Infarction – Intraop
- Myocardial Infarction – Postop
- Organ/Space SSI
- Pneumonia
- Pulmonary Embolism
- Sepsis
- Septic Shock
- Severe Sepsis
- Stroke/CVA
- Superficial Incisional SSI
- Transfusions w/in first 72 hours postop
- Unplanned Intubation – Postop
- Urinary Tract Infection – CAUTI
- Urinary Tract Infection – SUTI

Serious Complication

- AKI w/dialysis
- Anastomotic leak (excluding no intervention or abx intervention)
- Cardiac Arrest req. CPR – Intraop
- Cardiac Arrest req. CPT - Postop
- Deep Incisional SSI
- Myocardial Infarction – Intraop
- Myocardial Infarction – Postop
- Organ/Space SSI
- Septic Shock
- Severe Sepsis
- Stroke/CVA
- Unplanned Intubation – Postop

© 2019 Howard R et al. *JAMA Network Open.*
